# Adopted Children and State Control

**Published:** 1920-07-17

**Authors:** 


**THE PUBLIC WELL-BEING**—(*continued*)

### Adopted Children and State Control.

Some time ago a conference of the associated societies for the care and maintenance of infants appointed a representative and influential committee, under the Presidency of the late Adeline Duchess of Bedford, to examine the principle and practice of child adoption. After hearing a good deal of evidence on the subject they have now issued their report, which not only lays stress on the danger of adoption, but in its general tone seems to be opposed to both the principle and the practice, and explicitly declares that "the adoption of children should be practised only in exceptional cases, in which the separation of mother and child seem desirable for the future welfare of the child or when such adoption is practically inevitable."

No one is likely to quarrel with the general proposition that the maintenance of natural ties and family life is essential to the well-being of the community, but it is impossible not to realise that one of the consequences of the war has been to cause a great change in the old order by the destruction of family life through lack of houses and other causes, and that the number of children whom it is a perplexing problem to "place" is increased by the number of widows left with young families, quite apart from the question of illegitimacy. In these circumstances "exceptional cases" have become so pressing and numerous that they are indeed an important factor in the method of finding secure and comfortable homes for children. It

**THE PUBLIC WELL-BEING—(*continued*).**

seems to us rather singular that the committee, while deprecating in one sentence the formation of voluntary associations for promoting or effecting adoptions, suggest in another that the adoption of a child is such a serious step that it should be regulated by statute, and advocate that "the relative rights and liabilities of the parties to the transaction and of the adopted child should be defined, and all adoptions should require the sanction of some judicial authority and be officially recorded." We are heartily in agreement with a guarantee such as is here proposed, but surely it in itself would be a safeguard sufficient to obviate the necessity of extinguishing "officially registered" voluntary associations. Why could not the statutory regulation and the voluntary association exist side by side and work in harmony? The thing to be remembered is that there are thousands of children living under miserable conditions, in which they can thrive neither physically nor morally, for whom adoption may be the only means of escape. Evidence was given in behalf of two well-known child-adoption associations which seem to be doing excellent work and to be exercising great care in the selection of cases considered suitable for adoption. One of these, the National Child Adoption Association, has been asked up to the present to arrange adoptions for 7,000 children, of which 2,000 have been accepted. The National Adoption Society did not start work until January 1919. In that year 536 adopters applied, of whom 299 were passed as suitable, but in two months no fewer than 1,000 applications came from mothers to have their children adopted. It is a curious fact that the Society has received many inquiries from coalminers in Wales willing to become adopters. It would be interesting to know why this is so, and why the Society "has made a rule not to send children to that district."

